# Effect of osteopontin on TIMP-1 and TIMP-2 mRNA in chondrocytes of human knee osteoarthritis *in vitro*

**DOI:** 10.3892/etm.2014.1750

**Published:** 2014-05-29

**Authors:** FANG-JIE ZHANG, WEN-BO YU, WEI LUO, SHU-GUANG GAO, YU-SHENG LI, GUANG-HUA LEI

**Affiliations:** 1 Department of Orthopaedics, Xiangya Hospital, Central South University, Changsha, Hunan 410008, P.R. China; 2 Department of Orthopaedics, The First People’s Hospital of Nanpin, Nanpin, Fujian 353000, P.R. China; 3 Orthopaedics Institute of Central South University, Changsha, Hunan 410008, P.R. China

**Keywords:** osteoarthritis, tissue inhibitors of metalloproteinase-1, tissue inhibitors of metalloproteinase-2, osteopontin, chondrocytes

## Abstract

Tissue inhibitors of metalloproteinases (TIMPs) regulate the activity of matrix metalloproteinases (MMPs) and enzymes from the a disintegrin and metalloproteinase domain with thrombospondin motifs family in osteoarthritis (OA). Elevated osteopontin (OPN) levels in plasma, synovial fluid and articular cartilage are associated with progressive OA joint damage; however, the role of OPN in the pathological changes of knee OA remains undetermined. The present study was undertaken to examine the effect of OPN on the expression of TIMP-1 and TIMP-2 mRNA in chondrocytes from 16 patients with knee OA. In this study, following the stimulation of human chondrocytes with recombinant human OPN (rhOPN; 100 ng/ml and 1 μg/ml, respectively) for 48 h, MTT assay was used to determine cell viability while the quantitative polymerase chain reaction (PCR) was used to detect the alterations in TIMP-1 and TIMP-2 levels. The results illustrated that neither 100 ng/ml nor 1 μg/ml rhOPN caused cytotoxicity or apoptosis of chondrocytes and that the relative mRNA expression of TIMP-1 and TIMP-2 was significantly increased in the 1 μg/ml rhOPN group compared with that in the control group (P=0.022 and P=0.003, respectively). However, no significant difference in expression was revealed between the 100 ng/ml rhOPN and control groups (P=0.998 and P=0.209, respectively). In conclusion, OPN may have a protective effect against pathological changes in advanced-stage OA.

## Introduction

Osteoarthritis (OA), the most prevalent joint disease, is characterised by the progressive breakdown of articular cartilage. Cartilage is composed of two main extracellular matrix (ECM) macromolecules: Type II collagen and aggrecan, a large aggregating proteoglycan (1,2). Two main enzyme families are believed to be responsible for cartilage destruction in OA: The matrix metalloproteinases (MMPs) mediate cartilage collagen breakdown, whereas enzymes from the a disintegrin and metalloproteinase domain with thrombospondin motifs family mediate loss of cartilage aggrecan. Tissue inhibitors of metalloproteinases (TIMPs) regulate the activity of these enzymes (3). A balance between MMPs and TIMPs is necessary for the physiological processes of OA (4). In a previous study, incubation with human recombinant TIMP-1 or TIMP-2 inhibited >90% of MMP activity in conditioned media from porcine chondrocytes cultured in the presence of interleukin-1α (5).

Osteopontin (OPN) is a 44–75 kDa multifunctional phosphoprotein secreted by numerous cell types, including osteoclasts, macrophages, lymphocytes and epithelial cells. In a previous study, OPN mRNA isolated from human OA cartilage showed enhanced expression as compared with that in normal cartilage, and human OA chondrocytes exhibited upregulated levels of OPN (6). Furthermore, OPN was found to be expressed in bone forming cells and hypertrophic chondrocytes of the embryonic epiphyseal growth plates (7). Elevated OPN levels in plasma, synovial fluid and articular cartilage are associated with progressive joint damage in patients with knee OA. OPN could be predictive of prognosis with respect to the progression of knee OA, by serving as a biochemical marker for the determination of disease severity (8,9). However, the role of OPN in the pathological changes of knee OA remain undetermined.

The association between TIMPs and OPN levels in chondrocytes has never been previously reported in the literature, to the best of our knowledge. The purpose of the present study was to investigate the effect of OPN on TIMP-1 and TIMP-2 mRNA in chondrocytes of human knee OA *in vitro* to reveal the role of OPN in OA.

## Materials and methods

### Cultures of chondrocytes

The study protocol was consented by the patients and approved by the Institutional Review Board of the Xiangya Hospital, Central South University (Changsha, China). Articular hyaline cartilage tissue was removed from the tibial surfaces of 16 patients with knee OA who underwent total knee replacement. Subsequent to being washed twice with phosphate-buffered saline (PBS), cartilage was minced with a scalpel blade into 1–5 mm^3^ pieces, then cartilage slices were digested with 5–8 ml 0.2% collagenase II (Sigma-Aldrich, St. Louis, MO, USA) for 12–16 h at 37°C with 5% CO_2_. The digestion was terminated with 8–10 ml Dulbecco’s modified Eagle’s medium/F12 (DMEM/F12) (Hyclone, Logan, UT, USA). The released chondrocyte pellets at the bottom of the centrifuge tube were removed by suction and transferred to a culture flask following centrifugation at 150 × g for 6 min. Cells were then counted using a hemacytometer and cell viability was determined using trypan blue exclusion. Cell pellets were resuspended in 5 ml DMEM/F12 containing 15% foetal bovine serum (FBS; Gibco-BRL, Grand Island, NY, USA) and 1% penicillin/streptomycin solution (Gibco-BRL) and incubated for 24 h at 37°C with 5% CO_2_ in a plastic culture flask, following which non-adherent cells were washed out. The remaining adherent cells were cultured for an additional 2 weeks in a flask and the growth medium was changed every 3 days prior to trypsinization, and then passed to new culture flasks. Cell passages one through two were used for experiments.

### Cell treatment

For all of the experiments, chondrocytes were plated in six-well culture plates and serum-starved for 24 h in DMEM/F12 containing 1% FBS to synchronise cells in a non-activating and non-proliferating phase. Chondrocytes were then cultured in DMEM/F12 containing 15% FBS and divided into three groups: i) The control group, maintained as unstimulated and untreated controls; ii) the 100 ng/ml recombinant human osteopontin (rhOPN; 1433OP, R&D Systems, Minneapolis, MN, USA) group, treated for 48 h, iii) the 1 μg/ml rhOPN group, treated for 48 h.

### Cell viability assay

Cell viability following treatment with rhOPN for 48 h was determined by the colourimetric MTT assay. One day before rhOPN treatment, the cells were seeded into 96-well plates. After 48 h of rhOPN treatment, culture medium was removed and 20 μl MTT solution (5 mg/ml in PBS) was added into each well and incubated at 37°C with 5% CO_2_ for 4 h. The supernatant was then aspirated and the formazan reaction products were dissolved by dimethyl sulphoxide (Sigma-Aldrich) solution and agitated for 15 min. The spectrophotometric absorbance was measured on an ELISA plate reader at 570 nm by a Multiskan MK3-Thermo labsystems (Thermo Fisher Scientific, Inc., Waltham, MA, USA).

### Total RNA isolation, quantification and reverse transcription

Following treatment, chondrocytes were lysed and total RNA was extracted with TRIzol^®^ reagent (Invitrogen Life Technologies, Rockville MD, USA) according to the manufacturer’s instructions. Total RNA was quantified by spectrophotometry. Total RNA (1 μg) was converted to cDNA using the Revert Aid™ First Strand cDNA Synthesis kit (Fermentas, Thermo Fisher Scientific Inc.). In brief, 1 μg template RNA and 1 μl oligo (dT)_18_ primer were mixed gently with nuclease-free water to a total volume of 12 μl, centrifuged briefly and incubated at 65°C for 5 min. This was then chilled on ice, spun down and the vial was placed back on ice. A total of 4 μl 5× Reaction Buffer, 1 μl RiboLock™ RNase Inhibitor (20 U/μl), 2 μl 10 mM deoxyribonucleotide triphosphate mix and 2 μl RevertAid™ Moloney Murine Leukaemia Virus Reverse Transcriptase (200 U/μl) were added to compose a volume of 20 μl. This reaction was then incubated for 60 min at 42°C, following which the reaction was terminated by heating at 70°C for 5 min. The finished cDNA products were stored in aliquots at −80°C until required.

### Quantitative polymerase chain reaction (qPCR) assays

The primers were synthesised by Shanghai Sangon Bioengineering Corporation (Shangai, China) and were as follows: TIMP-1 forward, 5′-CCCAGAGAGACACCAGAGAAC-3′; and reverse, 5′-CACGAACTTG-GCCCTGAT-G-AC-3′; TIMP-2 forward, 5′-GCACATCACCCTCTGTGAC-TT-3′; and reverse, 5′-AGCGCGTGAT CTT GCACT-3′; β-actin forward, 5′-GGAAATCGTGCGTGACATTA-3′; and reverse, 5′-GGA GCAATGATCTTGATCTTC-3′.

A total of 12.5 μl Maxima^®^ SYBR Green/ROX qPCR Master Mix (2X; Fermentas), 2.5 μl forward primer (0.3 μM), 2.5 μl reverse primer (0.3 μM), 2 μl template DNA and 5.5 μl nuclease-free water added to a volume of 25 μl were used in all qPCRs. In addition, the ABI 7900 Sequence Detection System (Applied Biosystems, Foster City, CA, USA) was used for all qPCRs. The PCR thermal protocol consisted of 2 min at 50°C for uracil DNA glycosylase pre-treatment, then one cycle for 10 min at 95°C for initial denaturation, followed by 40 repeats of a 15-sec denaturation step at 95°C, a 30-sec annealing step at 30°C and a 30-sec extension step at 72°C. A melting curve analysis was performed subsequent to a final amplification period via a temperature gradient from 95°C for 15 sec to 60°C for 15 sec and 95°C for 15 sec.

### qPCR test results

The relative expression of mRNA was calculated as the ratio to the expression of β-actin mRNA, as a reference gene. Relative expression of genes of interest were calculated and expressed as 2^−ΔΔCT^. All quantities were expressed as n-fold relative to a calibrator.

### Statistical analysis

All results were analysed by SPSS 17.0 (SPSS, Inc., Chicago, IL, USA) for statistical evaluation, and are presented as the mean ± standard error of the mean. Statistical significance was determined using the Student’s t-test; P<0.05 was considered to indicate a statistically significant difference.

## Results

### Effect of rhOPN on chondrocyte viability in vitro

Table I shows the MTT data as fraction ratios to the control. Following incubation for 48 h, no significant difference was observed between the cell viability in the control group (99.64±1.14%) and that in the 100 ng/ml rhOPN (98.95±1.02%) (P=0.097) and 1 μg/ml rhOPN (98.88±0.76%) (P=0.129) treatment groups. These results revealed that rhOPN did not suppress the survival of human chondrocytes *in vitro*.

### Effect of rhOPN on TIMP-1 and TIMP-2 mRNA expression of chondrocytes in vitro

As shown in Table II, the relative TIMP-1 mRNA expression showed no significant difference between the 100 ng/ml rhOPN (1.000±0.539-fold) and the control group (P=0.998); however, the difference in the relative TIMP-1 mRNA expression between the 1 μg/ml rhOPN (2.060±1.667-fold) and control groups showed statistical significance (P=0.022), and the difference in the relative TIMP-1 mRNA expression between the 1 μg/ml (2.060±1.667-fold) and 100 ng/ml (1.000±0.539-fold) rhOPN groups also indicated statistical significance (P=0.026).

As shown in Table III, the relative TIMP-2 mRNA expression showed no significant difference between the 100 ng/ml rhOPN (0.806±0.590-fold) and the control group (P=0.209); however, the difference in the relative TIMP-2 mRNA expression between the 1 μg/ml rhOPN (1.891±1.198-fold) and the control group showed statistical significance (P=0.003), and the difference in the relative TIMP-2 mRNA expression between the 1 μg/ml (1.891±1.198-fold) and 100 ng/ml (0.806±0.590-fold) rhOPN groups also indicated statistical significance (P=0.004).

## Discussion

Cartilage damage is one of the main pathological changes in OA. In a previous study, Attur *et al* (10) revealed that the expression of OPN mRNA was highly upregulated in human OA cartilage as compared with that in normal cartilage. In the same study, it was also found that the spontaneous production of nitric oxide and prostaglandin E2 was suppressed under *ex vivo* conditions, following the addition of recombinant OPN to human cartilage affected by OA. These findings suggest that OPN is overexpressed in OA cartilage and functions as an endogenous inhibitor of the production of inflammatory mediators in cartilage. OPN deficiency exacerbates aging-associated and instability-induced OA, and both structural changes and an increased loss of proteoglycan from cartilage tissue have been observed to be augmented in the absence of OPN. OPN deficiency has also been shown to lead to the induction of MMP-13 (11). This indicates that OPN is involved in OA progression, although its role remains undetermined.

Cartilage degradation in OA is believed to be the consequence of an imbalance between MMP activity [collagenases (MMP-1 and 13), gelatinases (MMP-2 and MMP-9) and stromelysin (MMP-3)] and that of their corresponding inhibitors, the TIMPs (12). Of the three major MMPs that degrade native collagen (MMP-1, MMP-8, and MMP-13), MMP-13 has been suggested to be the most important in OA due to its preferential degradation of type II collagen (13). Furthermore, the expression of MMP-13 in OA has been observed to be significantly enhanced (14). The development of structure-modifying treatments for OA may focus on the inhibition of the synthesis and/or activity of MMPs. All active MMPs are inhibited by TIMPs. Among the different forms of TIMPs, TIMP-1 exhibits the highest affinity for MMP-1, MMP-13, MMP-3 and MMP-9 (15), while TIMP-2 forms a complex with type IV collagenase (16). TIMP-1 serum levels may serve to predict the prognosis of patients with hip OA (17). The initial concentration of TIMP-1, as well as the δ value of variation in serum levels of TIMP-1 (the difference between TIMP-1 concentration at entry and at the end), was shown to be correlated with the progression of joint space narrowing.

To the best of our knowledge, this was the first investigation to determine the effect of OPN on TIMP-1 and TIMP-2 mRNA in chondrocytes of human knee OA *in vitro*. The data from the MTT assay showed that the rhOPN did not suppress the viability of human chondrocytes *in vitro* after 48 h of incubation. Following stimulation with rhOPN for 48 h, the fold-change in TIMP mRNA expression relative to a normalised control group was measured, and β-actin was used as the endogenous control. The relative TIMP-1 and TIMP-2 mRNA expression was not revealed to be increased in the 100 ng/ml rhOPN group compared with that in the control group (P>0.05 for both) (Tables II and III). However, following stimulation with rhOPN at a concentration of 1 μg/ml for 48 h, the relative TIMP-1 and TIMP-2 mRNA expression was significantly increased compared with the control group (P<0.05 for both) (Tables II and III). Elevated OPN levels in the synovial fluid and articular cartilage are associated with disease severity in patients with knee OA (9); therefore, OPN may play an important role in the pathological changes in advanced-stage OA via promoting the expression of TIMP-1 and TIMP-2 to inhibit the degeneration of cartilage ECM activated by MMPs. The functions of OPN and TIMPs may have synergistic effects in OA due to the TIMPs inhibiting the levels of MMP-13 (15) and OPN deficiency also leading to the induction of MMP-13 in OA models (11). Paradoxically, in rheumatoid arthritis (RA), OPN produced by synovial fibroblasts in the synovial lining layer and at sites of cartilage invasion not only mediates attachment of these cells to cartilage, but also contributes to matrix degradation in RA by stimulating the secretion of collagenase 1 in articular chondrocytes (18). OPN may mediate bone resorption by osteoclasts in arthritis through ligation with its receptor, the α(v)β3 integrin. OPN may be a useful therapeutic target molecule in the prevention of bone destruction in arthritis (19).

In the present study, it was concluded that OPN may have a protective effect against the pathological changes in advanced-stage OA. Further studies are anticipated to clarify the pathways and to determine the different roles in OA and RA.

## Figures and Tables

**Table I:** Cell viability of each group.

Group	Chondrocyte viability *in vitro* (%)
Control	99.64±1.14
100 ng/ml rhOPN	98.95±1.02
1 μg/ml rhOPN	98.88±0.76

P>0.05, comparison among the groups. rhOPN, recombinant human osteopontin.

**Table II:** TIMP-1 mRNA levels of each group.

Group	Relative fold TIMP-1 mRNA level
Control	1
100 ng/ml rhOPN	1.000±0.539
1 μg/ml rhOPN	2.060±1.667

Results are presented as the mean ± standard error of the mean. Comparison between the 100 ng/ml rhOPN and control groups, P=0.998. Comparison between the 1 μg/ml rhOPN and control groups, P=0.022. Comparison between the 100 ng/rhOPN and 1 μg/ml rhOPN groups, P=0.026. TIMP-1, tissue inhibitor of metalloproteinases-1; rhOPN, recombinant human osteopontin.

**Table III:** TIMP-2 mRNA levels of each group.

Group	Relative fold TIMP-2 mRNA level
Control	1
100 ng/ml rhOPN	0.806±0.590
1 μg/ml rhOPN	1.891±1.198

Results are presented as the mean ± standard error of the mean. Comparison between the 100 ng/ml rhOPN and control groups, P=0.209. Comparison between the 1 μg/ml rhOPN and control groups, P=0.003. Comparison between the 100 ng/ml rhOPN and 1 μg/ml rhOPN groups, P=0.004. TIMP-2, tissue inhibitor of metalloproteinases-2; rhOPN, recombinant human osteopontin.
